# Multi-View Learning for Material Classification

**DOI:** 10.3390/jimaging8070186

**Published:** 2022-07-07

**Authors:** Borhan Uddin Sumon, Damien Muselet, Sixiang Xu, Alain Trémeau

**Affiliations:** 1 Univ Lyon, UJM-Saint-Etienne, CNRS, Institut Optique Graduate School, Laboratoire Hubert Curien UMR 5516, F-42023 Saint-Etienne, France; mdborhanuddin.sumon@etu.univ-st-etienne.fr (B.U.S.); alain.tremeau@univ-st-etienne.fr (A.T.); 2 Key Laboratory of Intelligent Perception and Image Understanding of the Ministry of Education of China, International Research Center of Intelligent Perception and Computation, School of Artificial Intelligence, Xidian University, Xi’an 710071, China; xusixiang@xidian.edu.cn

**Keywords:** material classification, multi-view learning, texture analysis, visual appearance, material dataset

## Abstract

Material classification is similar to texture classification and consists in predicting the material class of a surface in a color image, such as wood, metal, water, wool, or ceramic. It is very challenging because of the intra-class variability. Indeed, the visual appearance of a material is very sensitive to the acquisition conditions such as viewpoint or lighting conditions. Recent studies show that deep convolutional neural networks (CNNs) clearly outperform hand-crafted features in this context but suffer from a lack of data for training the models. In this paper, we propose two contributions to cope with this problem. First, we provide a new material dataset with a large range of acquisition conditions so that CNNs trained on these data can provide features that can adapt to the diverse appearances of the material samples encountered in real-world. Second, we leverage recent advances in multi-view learning methods to propose an original architecture designed to extract and combine features from several views of a single sample. We show that such multi-view CNNs significantly improve the performance of the classical alternatives for material classification.

## 1. Introduction

Material classification is a visual recognition task closely related to texture classification and dedicated to classifying input texture/material images into categories such as fabrics, wood, steel, or cotton [1]. It is of great interest to computer vision because predicting the material of objects in a scene can help for many applications: object manipulation by a robot [2], automatic waste sorting [3], predicting the appearance of an object under different lighting conditions [4], object recognition [5], etc.

**Figure 1:**
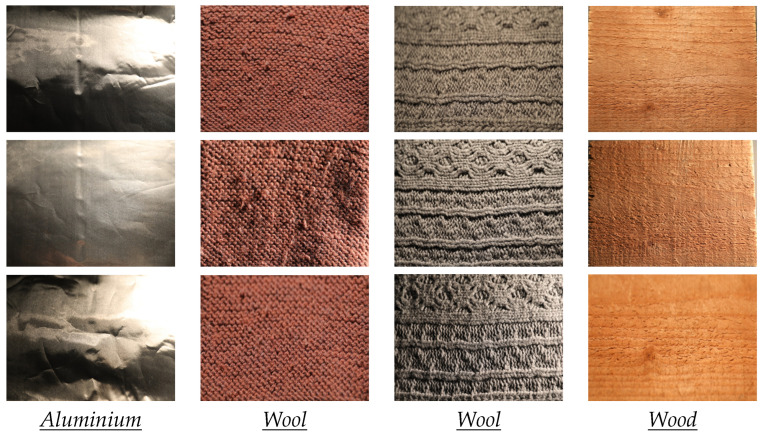
Appearance variation across acquisition conditions. The images of each column contain the same sample under different (lighting or viewpoint) conditions. These images are extracted from our new dataset.

However, this is still a challenging problem, since material images show a large intra-class variability [1,6]. First, the visual appearance of a material or a texture sample may significantly vary across viewing and lighting conditions. This is illustrated in Figure 1, where each column represents the same sample but observed under different lighting conditions and viewpoints. Second, different samples made from the same material can have different visual features, even when observed under similar conditions. This is the case, for example, with the two wool samples displayed in columns 2 and 3 of Figure 1. These two problems are very important for material recognition tasks and make it very challenging to extract relevant features from color images.

Recent studies have shown that deep neural networks clearly outperform many alternatives for material classification, but it is also clear that their performances are highly related to the data on which they are trained and tested [1,6,7]. For a material dataset showing small variations across acquisition conditions, a deep network can easily learn the specific features of each material and provide a very good recognition accuracy. When high variability exists in the acquisition conditions of the images (as for the real-world material appearance), we show, in this paper, that the performances can significantly drop. The first contribution of this paper is the constitution and provision of a dataset of material images with large intra-class variability; see Section 3.1. This dataset is called UJM-TIV (UJM is the abbreviation of our university, and TIV stands for Textures under varying Illumination, pose and Viewing). In this paper, we leverage this dataset to confirm that current classical neural network solutions do not generalize sufficiently to new data for real-world material observations. We hope that such a new diverse dataset will help to better learn material features in the future.

Then, in order to go a step further towards better generalization of deep features for material classification, we propose exploiting a multi-view learning solution. Indeed, since one image provides a single view of a material sample, we claim that the performance could be significantly improved by considering a set of images for each material sample. Indeed, when a human being tries to determine the material that constitutes an object, they often tend to vary their point of view by moving their head or manipulating the object when possible to vary the viewpoint and light direction. We propose to mimic this natural behavior by taking advantage of the recent advances in multi-view learning [8], which makes it possible to extract features from several images and to merge them into a relevant representation. To the best of our knowledge, this is the first time that a multiview learning approach is applied to material images in order to tackle the problem of appearance variations across viewing conditions.

Our contributions are fourfold:

- We analyze the current material datasets and show that they do not have enough intra-class diversity for material classification tasks,
- We provide a new public material dataset with high variations across acquisition conditions (lighting and viewpoint) in order to better represent the multiple appearances of a single real-world material sample,
- We propose to exploit a multi-view learning approach to extract features from a set of images of the same material sample and to merge them into an accurate material representation,
- Extensive tests on two material datasets show that exploiting multiple views of the same material sample clearly outperform the single-view alternative.

In Section 2, we present state-of-the-art solutions designed for material classification and multi-view learning and discuss the different public material datasets. Next, Section 3.1 is devoted to the description of our new dataset. We detail the materials used, the lighting conditions, the acquisition device, and the viewing conditions. We show why this dataset is more adapted for multi-view learning than the classical KTH-TIPS2 dataset [9] or any other existing datasets. The KTH-TIPS2 dataset can be downloaded from [10]. Next, in Section 3.2, we present a deep network architecture designed for two-view learning and test it on two material datasets, showing that it outperforms the alternative deep single-view classifier. The experimental results are reported in Section 4. Lastly, a conclusion is drawn and future research directions are indicated in Section 5.

## 2. Related Work

### 2.1. Material Classification

Several categories of methods have been proposed in state-of-the-art studies. The first ones are related to pattern-recognition-based methods, i.e., the computation of image features such as textons [11,12]. Next, methods based on filter banks have been proposed. These are related to the computation of local texture features [13,14,15,16,17]. Then, local texture features aggregation methods, such as the bags-of-textons [18], have been introduced, which are designed to compute global texture features.

Some recent papers have demonstrated the efficiency of CNN methods for material recognition (e.g., [19]) and the superiority of deep networks and off-the-shelf CNN-based features (e.g., [20]), particularly with non-stationary spatial patterns, such as textures, and in the presence of multiple changes in the acquisition conditions, against traditional, hand-crafted descriptors [1]. In [7], a selection of CNN architectures were evaluated and compared on various widely used material databases and achieved up to 92.5% mean average precision using transfer learning on the MINC 2500 material database. In [1], a selection of state-of-the-art solutions (LFV+FC-CNN [21], Deep Ten [22], FV-CNN [23], and B-CNN [24]) designed for material classification were evaluated and compared on various datasets (FMD, KTH-TIPS-2b, and 4D-Light). The best classification accuracy obtained with these networks was around 83% for only the KTH-TIPS-2b dataset.

Until recently, most material classification methods used only single-view image as input or combined few single view image features as input. For example, in [25], the authors used a multi-modal sensing technique, leveraging near-infrared spectroscopy and close-range high-resolution texture imaging, to perform material classification.

In [26,27], the authors demonstrated that the concept of photometric stereo acquisition could improve the efficiency of material classification methods. They showed how micro-geometry and reflectance properties of a surface could be used to infer its material. Likewise, Maximov et al. [28] and Vrancken et al. [29] demonstrated that combining different lighting and viewing conditions could slightly improve the material classification task.

In the ideal case, the user would like to predict the appearance of a material regardless of the viewing direction and other factors that could have an impact on the capturing process. This is a quite challenging, ill-posed, and under-constrained problem that remains hard to solve for the general case [6].

### 2.2. Multi-View Learning

The aim of multi-view learning is to extract accurate features from data of different modalities (color image, text, audio, Lidar, etc.), or representing different views of the same sample (different languages for texts, different acquisition conditions for images, etc.) [8].

Features can be extracted from images very accurately with convolutional neural networks (CNN), and many approaches have integrated multi-view learning into CNN [8,30,31,32]. The idea is to aggregate CNN features from different views into a more accurate general representation. Two main approaches based on multi-view CNN exist, as presented in [8]: the so called one-view-one-net mechanism uses one network per view and aggregates all the features through a fusion process [30,31], while the multi-view-one-net mechanism feeds a single network with all the views to extract features [32]. For the one-view-one-net solutions, the first networks used to extract the features usually share their weights in order to minimize the number of learned weights. The crucial points of such approaches lie in the feature-fusion process. The main question with the multi-view-one-net solutions is about the aggregation of the inputs images before feeding the single network. The straightforward approach consists in concatenating these images into a multi-channel image and to apply convolutions on this image. This means that local features are extracted at the same locations in these images, which requires a coarse registration between the images in order to obtain consistent features. Second, such a concatenation prevents the use of pre-trained networks that are usually fed with three-channel images. Therefore, in this paper, we have chosen a one-view-one-net approach with a specific architecture.

Finally, some approaches have also combined Siamese networks with multi-view learning for person re-identification [33] or image quality assessment [34], for example. Varga et al. propose extracting a set of overlapping sub-windows from a person image and feeding a Siamese network with these different views (sub-windows) [33], while Liang et al. also feed a Siamese network with sub-patches extracted from color images [34].

Even though each element of our designed network has been carefully selected, the contribution of this paper is not in the definition of a new architecture for a general multi-view CNN. The main aim is rather to show that multi-view learning is an appropriate solution to tackle material classification. To the best of our knowledge, this is the first time that a multi-view CNN has been used for this task.

### 2.3. Material Datasets

Several categories of texture/material datasets have been introduced over the years. Some image sets were collected in lab settings from cropped stand-alone samples (e.g., CUReT [35] in 1999, KTH-TIPS [36] in 2005); meanwhile, others were collected in the wild (e.g., FMD [37] in 2009, OpenSurfaces [38] in 2013, MINC [39] in 2015, and LFMD [40] in 2016) with more diverse samples and real-world scene contexts. The number of classes and the number of samples in each class varies greatly from one dataset to another (e.g., 10 classes/810 images in total for KTH-TIPS, 61 classes/5612 images in total for CUReT); likewise, the diversity of input parameters also varies significantly (e.g., small viewpoint changes in KTH-TIPS, larger viewpoint changes in CUReT) [41]. The KTH-TIPS (Textures under varying Illumination, Pose and Scale) image database was created to extend the CUReT database by providing variations in scale [36].

**Figure 2:**
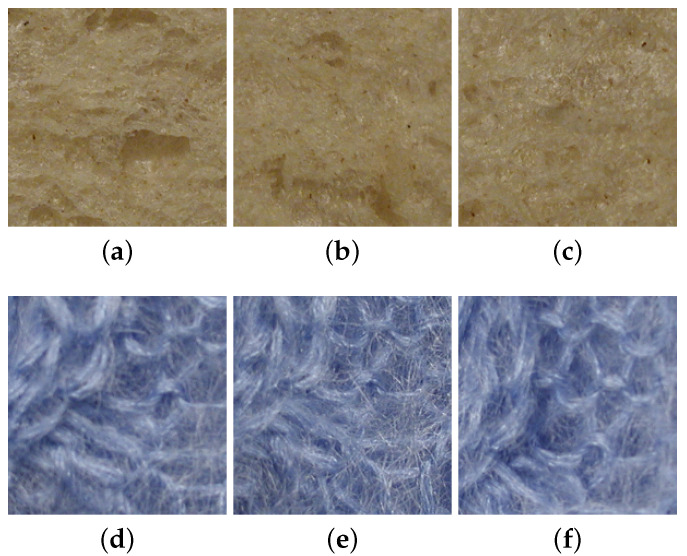
Changes in visual appearance of a white bread sample and a wool sample from the KTH-TIPS2 dataset under various lighting and viewing directions. Images (**a**–**c**) were captured with a frontal illumination direction and frontal, 22.5° right and 22.5° left viewing directions, respectively, for the white bread sample. Similarly, images (**d**–**f**) were captured with a frontal illumination direction and frontal, 22.5° right and 22.5° left viewing directions, respectively, for a wool sample.

**Figure 3:**
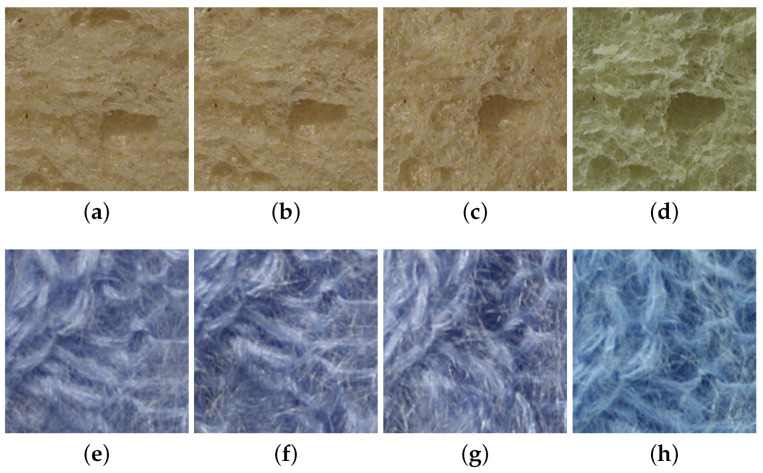
Changes in visual appearance of a white bread sample and a wool sample from the KTH-TIPS2 dataset under various lighting and viewing directions. Images (**a**–**d**) were captured with a frontal viewing direction and frontal, 45° from the top, 45° from the side, and ambient illumination conditions, respectively, for a white bread sample. Similarly, images (**e**–**h**) were captured with a frontal viewing direction and frontal, 45° from the top, 45° from the side, and ambient illumination conditions, respectively, for a wool sample.

KTH-TIPS2 is an extension of the KTH-TIPS [9] database. KTH-TIPS2 contains 4 physical samples of 11 different materials (the same material classes as KTH-TIPS) [42]. Similarly to the KTH-TIPS dataset, it provides planar images with variations in scale, as well as variations in pose and illumination. From one physical sample to another one, there is in some classes some strong (intra-class) variations (e.g., within wool or cracker samples); meanwhile, for some other classes, intra-class variations are lower (e.g., within wood or cork samples). There is also some similitude between cotton and linen classes (i.e., a small inter-class variance). In CUReT, only a single material instance is provided per class; consequently, no generalization can be performed to classify material categories due to a lack of intra-class variation. Changes in KTH-TIPS2 induced by a change in viewing directions or by a change in lighting conditions are, respectively, illustrated in Figure 2 and Figure 3.

In most material datasets, the viewing and lighting conditions and the camera settings are well controlled, and image acquisition is performed by a technician (a photographer) who takes care to perform the best acquisition (e.g., to minimize the blur and to minimize specularity) with the available setup system. However, for some materials, such as aluminum foil samples, this is very challenging as this kind of material is very reflective.

**Figure 4:**
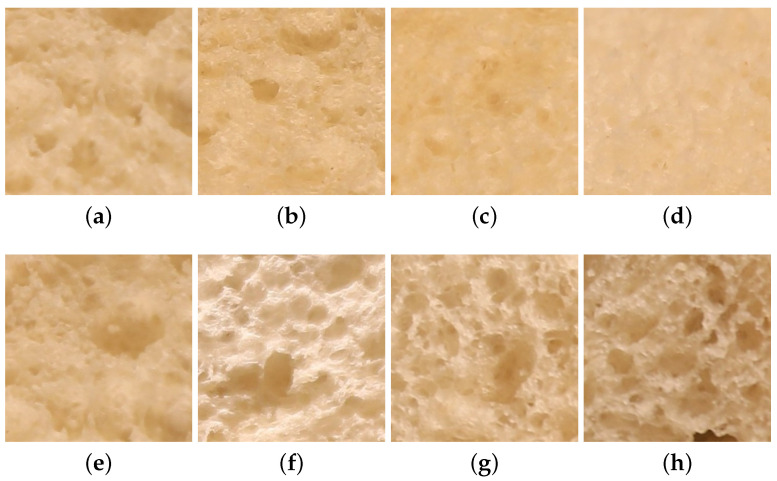
Changes in the visual appearance of a white bread sample under various lighting geometries and viewing directions. Images (**a**–**d**) were acquired under the same lighting direction (90°). Images (**e**–**h**) were acquired under the same viewing direction (90°). For images (**a**–**d**) the lighting direction was fixed at 90° and the viewing directions are 90°, 60°, 35°, and 10°, respectively. For the images (**e**–**h**), the viewing direction is fixed at 90° and the lighting directions are 90°, 65°, 45°, and 20°, respectively.

Our aim was therefore to create a new dataset giving greater flexibility to the user in the image-acquisition process. Our main objective was to perform image acquisition under various lighting and viewing directions, rather than under very strict and well-controlled (and limited) lighting and viewing conditions. We assume that from one viewing direction to another one, the average lightness of the sample may differ, as illustrated in Figure 4f, in comparison with Figure 4h. Lightness/color invariance is one of the invariance properties that a material classifier should have. We also assume that from one viewing direction to another one, the contrast of the sample may differ, depending of the roughness and thickness of the materials, as illustrated in Figure 4a in comparison with Figure 4e. Contrast invariance is one of the invariance properties that a material classifier should also have.

The fabric dataset introduced in [27] illustrates another kind of lightness shift due to a lighting field (an array of 12 LEDs) that is not spatially uniform on the sample area. This dataset contains 1266 samples that belong to one of the following fabric classes: cotton, terrycloth, denim, fleece, nylon, polyester, silk, viscose, and wool. The number of samples in each class is very unbalanced (588 in the cotton class, 32 in the terrycloth class). The samples were acquired under near-grazing illumination from a frontal view only. To perform photometric reconstruction, the setup was geometrically calibrated.

**Figure 5:**
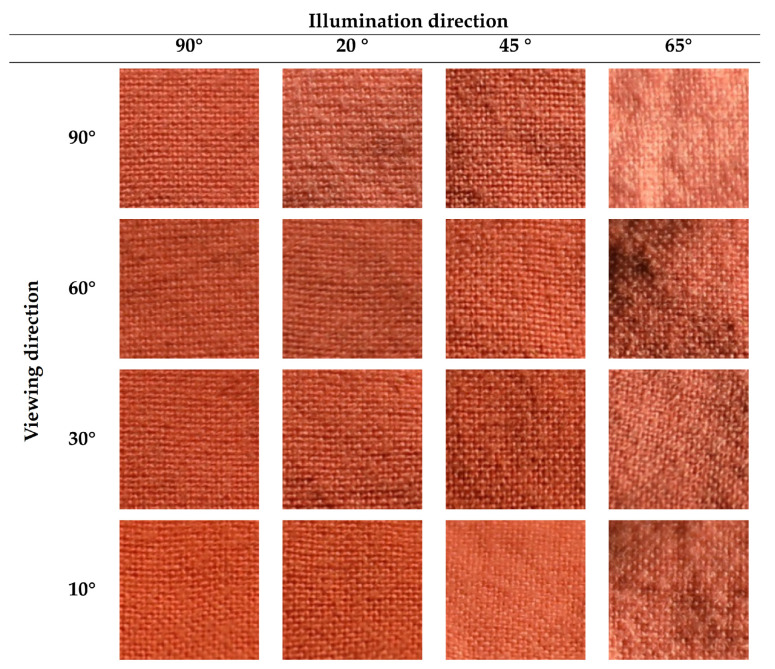
Images of a cotton sample from the UJM TIV dataset observed under different views.

**Figure 6:**
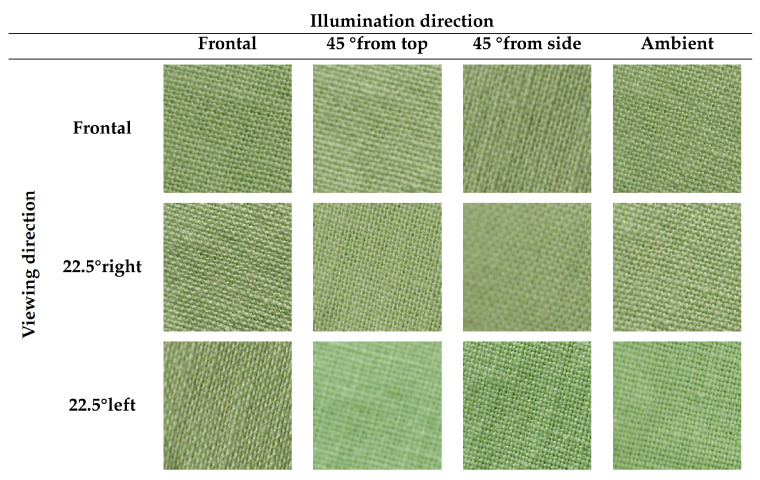
Images of a cotton sample from the KTH-TIPS2 dataset observed under different views.

By playing with lighting and viewing conditions, we can increase the difference in the visual appearance for a material sample. In this paper, we claim that the diversity of the visual appearances of a material sample over variations in acquisition conditions should be accounted for in the final feature vector to optimize the classification accuracy. For example, the image differences observed in Figure 5 are more significant than those observed in Figure 6, as higher viewing and lighting angles were considered in the UJM-TIV dataset than in the KTH-TIPS2 dataset (see complementary information provided in Table 1 and Table 2).

In next section, we present the details of our new datasets and the way we propose to exploit multiple views of a single material in order to boost the classification performance.

## 3. Materials and Methods

### 3.1. Our New Material Dataset: Ujm-Tiv

#### 3.1.1. General Comments

**Figure 7:**
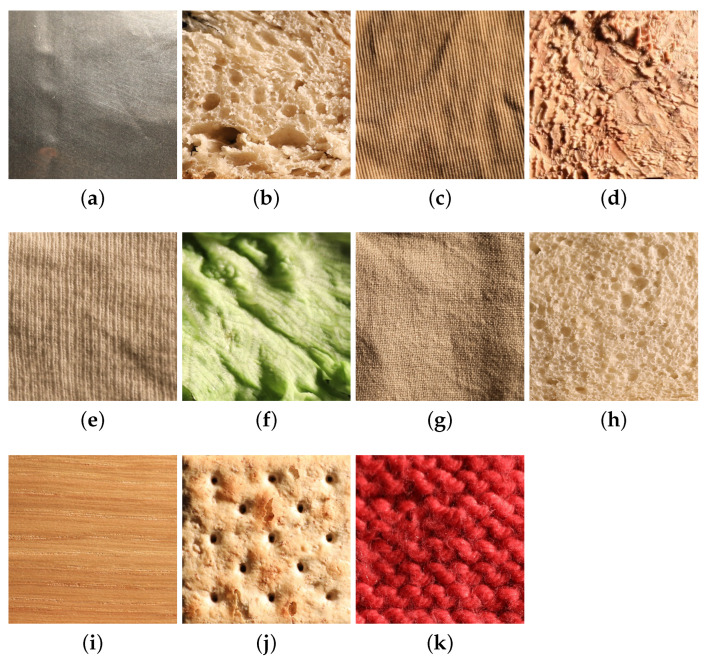
Images of samples of (**a**) aluminium foil, (**b**) brown bread, (**c**) corduroy, (**d**) cork, (**e**) cotton, (**f**) lettuce leaf, (**g**) linen, (**h**) white bread, (**i**) wood, (**j**) cracker, and (**k**) wool from the UJM-TIV dataset taken under illumination conditions of 65° and viewing condition 90°.

The UJM-TIV material dataset consists of images from 11 distinct classes, namely aluminium foil, brown bread, corduroy, cork, cotton, lettuce leaf, linen, white bread, wood, cracker, and wool (see Figure 7).

**Figure 8:**
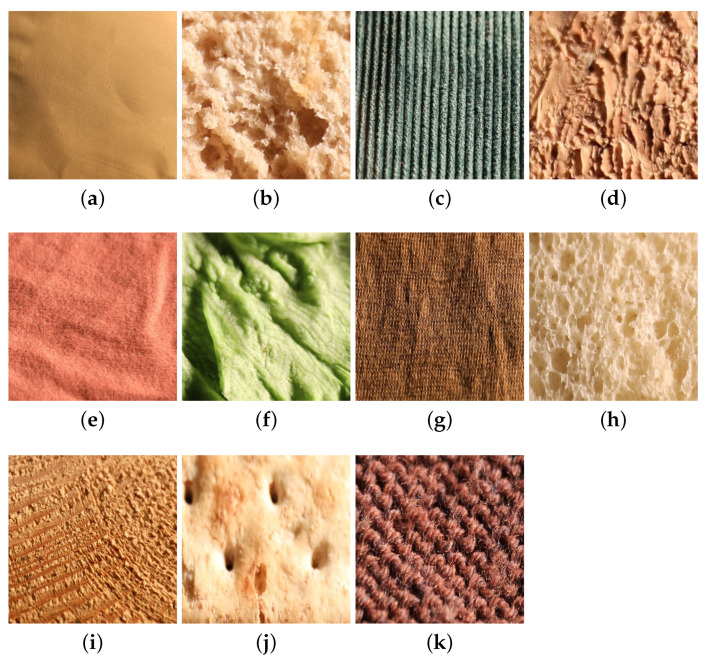
Images of a sample of (**a**) aluminium foil, (**b**) brown bread, (**c**) corduroy, (**d**) cork, (**e**) cotton, (**f**) lettuce leaf, (**g**) linen, (**h**) white bread, (**i**) wood, (**j**) cracker, and (**k**) wool category from the UJM-TIV dataset taken under a illumination direction of 65° and a viewing condition of 35°.

These images were acquired under controlled viewing and lighting conditions. These 11 classes are also included in the KTH-TIPS2 [42] dataset. Due to the diversity of samples in each material category, the visual appearance of the UJM-TIV samples is not similar to that of the KTH-TIPS2 samples. Strong differences in appearance with respect to Figure 7 are evident at lower viewing angles or lower illumination angles (see Figure 8).

**Figure 9:**
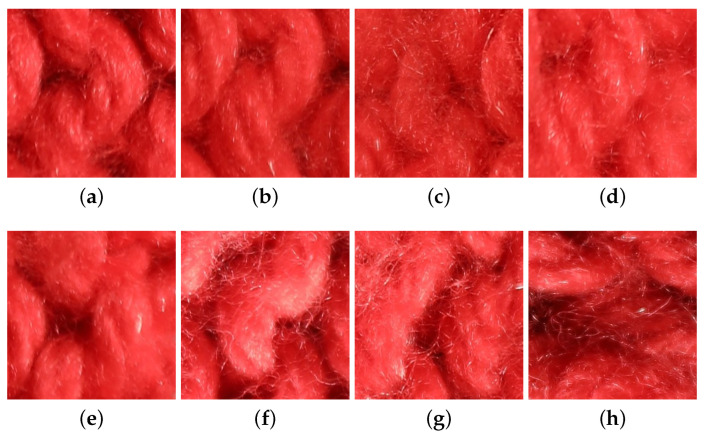
Changes in visual appearance of a wool sample under various lighting geometries and viewing directions. Images (**a**–**d**) were acquired under the same lighting direction (90°). Images (**e**–**h**) were acquired under the same viewing direction (90°). For images (**a**–**d**), the lighting direction is fixed at 90°, and the viewing directions are 90°, 60°, 35°, and 10°, respectively. For images from (**e**–**h**), the viewing direction is fixed at 90° and the lighting directions are 90°, 65°, 45°, and 20°, respectively.

In the UJM-TIV dataset the variation in appearance between samples is clearly larger for some categories (e.g., wood and wool) than in KTH-TIPS2. Furthermore, in UJM-TIV, wool and cotton have the highest variations in appearance, while cork, brown bread, and white bread have the lowest intra-class variations. As an illustration, see the changes in appearance shown in Figure 1 and Figure 9.

#### 3.1.2. Acquisition Settings and Image Processing

**Figure 10:**
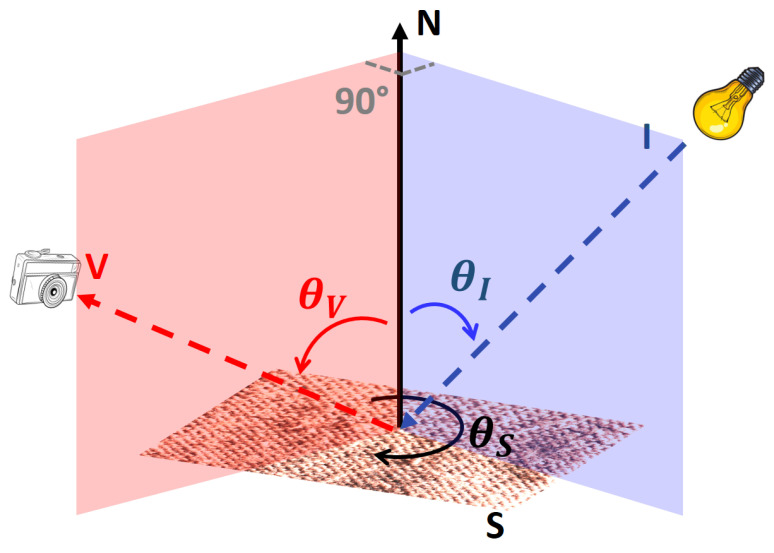
Schematic diagram of the image acquisition setup. In our experiments, the plane defined by vectors N and I was set perpendicular to the plane defined by vectors N and V.

For our dataset, a Canon EOS 5D Mark IV digital camera was used to capture the images of the samples with a resolution of 6720 × 4480 pixels. The background surrounding each sample was removed using a post-processing step. For each object sample, two object poses were considered, with a 90°rotation around the surface normal N of the angle denoted $\theta_{S}$ in Figure 10.

**Figure 11:**
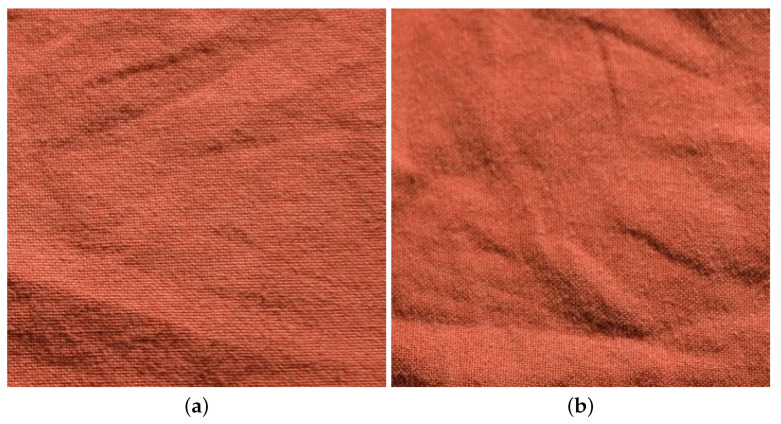
Images of a cotton sample from UJM-TIV: (**a**) when the viewing condition is frontal and lighting condition is at 20°. (**b**) with the same viewing and lighting conditions when the sample orientation is perpendicular.

The example shown in Figure 11 illustrates how such a change can modify the material appearance for a given material sample.

The image acquisition setup used to capture the images under controlled viewing and lighting conditions is illustrated in Figure 10. In this Figure, S is the material sample, I is the illumination source, and V is the viewpoint direction. The plane defined by the vectors N and V is perpendicular to the plane defined by the vectors N and I. Four standard light sources (60 W tungsten light bulb) were used, one for each lighting direction $\theta_{I}$ (frontal, roughly 20°, roughly 45°, and roughly 65°). Four viewing directions $\theta_{V}$ (frontal, roughly 60°, roughly 30°, and roughly 10°) were used for each object pose. Therefore, there is a total of 16 (four illumination directions x four viewing directions) images per sample position captured for each material sample. For two poses, a total of 32 images were captured for each sample. The acquisition were performed in a dark room without any ambient illumination.

The Patchify [43] library was used to extract 200×200 pixel image patches from the samples. Areas with a background and too blurry images were removed manually from all extracted patches. The number of patches extracted varied from sample to sample. The dataset contains around 75 thousand image patches after areas that were blurred and out of focus were removed from the all extracted patches.

#### 3.1.3. Comparison with Previous Datasets

The viewing directions used in UJM-TIV are different from those used in KTH-TIPS2 (frontal, rotated 22.5° left and 22.5° right) and with a larger range. The lighting directions used in UJM-TIV are also different from those used in KTH-TIPS2 (frontal, 45° from the top and 45° from the side, all taken with a desk-lamp with a Tungsten light bulb).

All samples captured in the KTH-TIPS2 were acquired under a combination of three viewing directions (frontal, rotated 22.5° left, and rotated 22.5° right) and four illumination directions (from the front, from the side at roughly 45° and from the top at roughly 45°, and using ambient lighting), unlike the ones used in UJM-TIV. They were also captured at different scales, which is the opposite of UJM-TIV.

**Figure 12:**
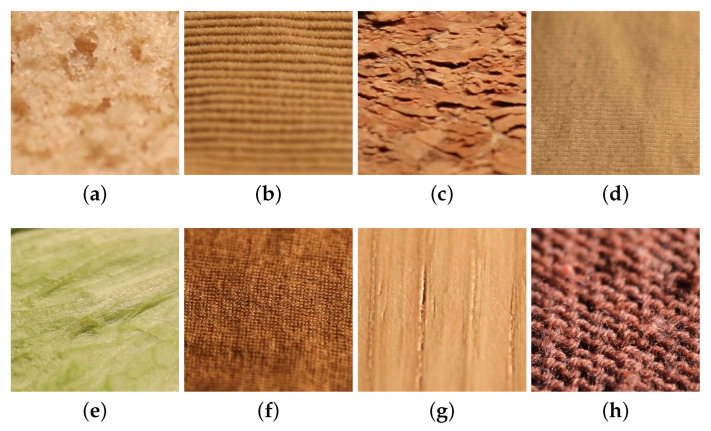
Image samples appeared as out of focus for the categories (**a**) brown bread, (**b**) corduroy, (**c**) cork, (**d**) cotton, (**e**) lettuce leaf, (**f**) linen, (**g**) wood, and (**h**) wool from the UJM-TIV dataset.

As with KTH-TIPS2, in UJM-TIV, few images of fine-structured materials appear out of focus at working distances due to perspective effects and roughness of materials; see Figure 12, where all the images shown were captured under a viewing direction around 10° and an illumination direction of 20°.

In contrast with other setups, such as the ones described in [27] or [44], in this study, our aim was not to tailor a lighting system that optimizes the light source positions depending on the various materials.

### 3.2. Multi-View Learning with Siamese Networks

Multi-view learning is attracting many researchers today [8] since it allows one to extract features from multiple views and to merge them into an accurate global representation. As explained above, a one-view-one-net mechanism is well adapted to material classification. In this case, each image (view) is fed into a deep backbone to extract features, and then the features of each view are merged and used as input to a classification network that predicts the class of the considered sample. Once again, our contribution, here, is not in the definition of the best architecture for this task but rather to leverage the multi-view learning area to show that it can significantly improve the performance for material classification.

Hence, we have selected a simple one-view-one-net architecture with a pre-trained network, leaving for future works any improvements related to the architecture choice.

Since each view feeds a backbone, we propose sharing the weights between these backbones in order to minimize the number of learned weights and to prevent overfitting. Furthermore, sharing the weights between backbones can also help to improve the generalization power of the model, since the same backbone must extract accurate features from different views (different appearances). A single architecture merging the outputs of two identical branches is a Siamese network [45,46,47].

**Figure 13:**
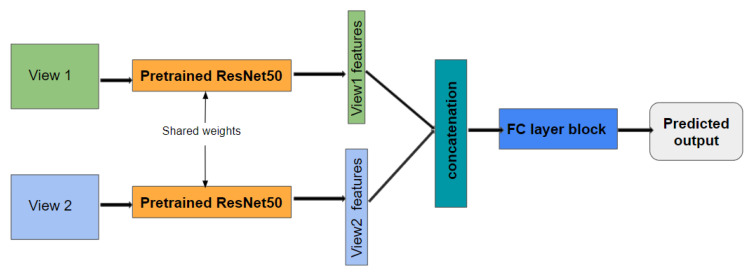
The proposed Siamese architecture for multi-view learning.

The architecture of the proposed network is shown in Figure 13. The Siamese network takes a pair of images as input from two different views and feeds it to one backbone. In our case, a pre-trained ResNet50 [48] is used as the backbone. Each branch learns the features from each input view. Then, the learned features are concatenated together, and the result feeds the fully connected layers for classification. It is worth mentioning that all the blocks are differentiable so that this architecture can be trained end-to-end (feature extraction and classification) with a single classification loss.

Before concatenating the features of each view, a global average pooling (GAP) layer is used in order to reduce the number of inputs of the first fully-connected (FC) layer of the architecture. It is known that such pooling helps to prevent overfitting problems [48].

This GAP layer averages all the local neuron activations into a single activation for each channel. One alternative would be to use a global max pooling (GMP) layer that preserves only the highest score over the activation map. Intuitively, GAP is designed to work on repetitive local patterns, where the average of similar features has a meaning and noise is removed, while GMP is designed to pick the most important detail in each map. In this case, we believe that, for texture images (with repetitive patterns), GAP is more appropriate than GMP.

Furthermore, in order to regularize the classifier, dropout is applied in the FC layers.

The advantage of such an architecture is that it can be easily adapted to more than two views. Indeed, the pre-trained backbone can be used to extract features from any new views, and only the FC layer has to be adapted and retrained to perform classification. In this paper, we have only trained and tested a two-branch architecture.

## 4. Results and Discussion

In order to assess the quality of our new dataset and the performance of the proposed multi-view CNN, we have conducted many tests on two datasets. The idea was to compare the advantages of our dataset over the KTH-TIPS2 dataset and to compare the performance of our two-views CNN with a single-view alternative.

### 4.1. Experimental Settings

We have created two architectures for our tests. One is a classical single-branch architecture with a convolutional backbone to extract features and FC layers for classification. The accuracy provided by this network is called single-view accuracy. Then, we used our Siamese architecture with two backbones with shared weights that extract features from two views and FC layers for classification. This architecture provides the so-called multi-view accuracy. As the backbone for these architectures, we selected a residual network ResNet50 [48] pre-trained on the ImageNet dataset. The last convolutional layer of this network is fine-tuned on the considered data, while the other layers are frozen. For each architecture, the number of FC layers and the number of neurons in each layer are cross-validated for fair comparison. Finally, the number of learned parameters is equivalent between each architecture (7.1 million for the single-view and 7.7 million for the multi-view).

Likewise, the hyperparameters and optimization algorithms are the same for both networks. We use the Adam optimizer with an initial learning rate of 0.001. For each experiment, the learning rate automatically decreases by a factor of 0.2 when the loss does not decrease for some consecutive steps. The maximum number of epochs is fixed to 350. Input images were resized to 224×224 before feeding the network with a batch size of 16.

The Keras framework with TensorFlow 2.8.0 backend and Python version 3.9.5 was used to implement both the single-branch and the Siamese network. The models were trained on a high-performance GPU with an NVIDIA RTX 8000 8 GB graphics card, CUDA version 11.2, and RAM of size 16 GB.

### 4.2. Data

We ran experimental tests with two different configurations. The first configuration consists in training and testing on the whole considered dataset. Each dataset is randomly split into training and test sets, with 70% and 30% of the data, respectively, providing the sets called KTH-TIPS2 Train, KTH-TIPS2 Test, UJM-TIV Train, and UJM-TIV Test.

Then, in order to test the multi-view learning, we selected some views in both datasets: 12 views in KTH-TIPS2 and 16 views in UJM-TIV. All the images of each selected view were also randomly split at a ratio 70% and 30% for training and testing, respectively.

Table 1 details the viewing and illumination conditions of the selected views from the KTH-TIPS2 dataset. As observed in Figure 6, changes in viewing and illumination directions have an impact on the overall appearance of the observed cotton sample (more blur, less contrast, etc.), but these changes are not significant (lower than the changes in appearance between samples belonging to the same category, i.e., changes induced by intra-class variation).

Table 2 details the viewing and illumination conditions of the selected views from the UJM-TIV dataset as shown in Figure 5 used for the experiment. Similarly, Figure 4 shows the images of four different views of a white bread sample from our new dataset used in the multi-view experiment.

### 4.3. Results

The results are organized into two sections, depending on which data the networks have been trained and tested. First, we show results for test on the whole datasets and then, results on selected views.

#### 4.3.1. Appearance Diversity of the Datasets

First, the idea is to analyze the results of a single-branch network on the whole datasets. The results are provided in Table 3 for both datasets. First, we can notice that the obtained accuracy for KTH-TIPS2 (80%) is similar to the ones obtained by classical deep networks in [49]. Second, we notice that the accuracy obtained on our UJM-TIV dataset with the same settings as the ones used on KTH-TIPS2 is much lower. This means that a single-branch network performs better on KTH-TIPS2 than on our dataset. We think that it is directly related to the higher intra-class variability of our dataset.

#### 4.3.2. Multi-View Learning

In this section, we provide results on both datasets when the networks are trained and tested on the selected views. We consider the views by pairs in order to test our deep architecture for the two views. Thus, we have trained a network (single- or two- views) with the images of the two considered views (the training set) and tested on the same views (the test set).

The results are provided in Table 4 for the KTH-TIPS2 dataset and in Table 5 for our UJM-TIV dataset. First, we can notice that considering only two views for training overall reduces the accuracy compared when training on the whole dataset (which was 80% for KTH-TIPS2 and 55% for UJM-TIV). This is not surprising since here, the network has been trained on fewer data than when the whole dataset was used. Second, we observe that the multi-view network significantly outperforms the single-view network for all selected view pairs. This clearly shows that multi-view learning is a relevant solution for material classification. Furthermore, we can notice that the improvement provided by the multiview training over the single-view training is much higher when the two views present very different appearances. This is the case for the dataset KTH-TIPS2 between view9 and view10 (+46% improvement), where there is a difference of $45^{\circ }$ for the viewing direction between the two views. For our dataset, the improvement from a single view to two views is important for almost all the considered pairs of views. This is due to the high variation in appearance between the views for our dataset.

These results clearly show that our dataset is well designed to train networks for material classification and that the proposed Siamese architecture is a relevant solution for two-view learning.

**Figure 14:**
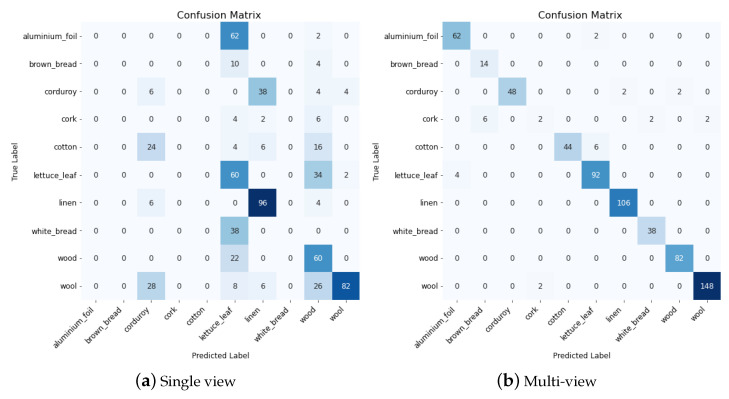
Confusion matrix for (**a**) Single-view model and (**b**) multiview model when using view5 and view6 from UJM TIV dataset.

To go a step further in the analysis, we propose looking at the confusion matrices for one experiment where the multi-view approach clearly outperforms the single-view, i.e., the tests on views 5 and 6 for the UJM-TIV dataset. These confusion matrices are displayed in Figure 14.

These matrices clearly show that many classification failures are avoided when multiple views are considered. Indeed, we can see in Figure 14a that many images are misclassified as a lettuce leaf or wood when using a single view, while most of the predictions are correct (diagonal) on Figure 14b.

#### 4.3.3. Experiments with a State-of-the-Art Solution

The previous experiments have shown that the results of a basic network not specifically designed for material classification can be strongly improved when considering a multi-view approach. As a final experiment, we checked if a very accurate state-of-the-art solution can also benefit from our contribution. Consequently, we have selected an approach adapted to material classification and based on the deep Fisher score [50]. This solution exploits orderless pooling and sparse coding and requires a training phase constituting three consecutive steps. We have trained and tested this network on all the view pairs presented in Table 4 and Table 5 in the context of single-view and multi-view learning. The results appear in Table 6 and Table 7.

These results confirm that the tested network is relevant for material classification, since it outperforms all the results from Table 4 and Table 5. Second, we can notice that, even with such an accurate network, moving from single-view to multi-view learning improves the results for almost all the experiments. Since the results of single-view classification are already very high, the relative improvements are much lower than in the previous case with a baseline network. Nevertheless, the average improvement on the KTH-TIPS2 dataset (Table 6) is significant (around 4.8%), and the results are almost perfect on the UJM-TIV dataset when combining the strong network from [50] and the proposed multi-view approach (Table 7). This last experiment clearly shows that our contribution can be exploited to boost the results of any state-of-the-art solution to the material classification task.

## 5. Conclusions

In this paper, we have proposed several contributions to material classification. We have introduced a new dataset with large intra-class variability. The variations in appearance within each class are due to large range of acquisition conditions and the selection of diverse material samples. We have shown that classical deep networks cannot easily generalize on such data, demonstrating the need for alternative solutions for this task. In order to exploit the appearance variations across viewing conditions, we have proposed leveraging the strengths of recent solutions in multi-view learning. We have shown that a Siamese architecture significantly outperforms the single-branch alternative by merging features from two views. Obviously, increasing the number of views at the input of the network is a solution that will be investigated in our future works. The challenge here is to extract features from uncontrolled views and to merge them into a general representation of the considered sample. Next, we plan to demonstrate that multi-view learning could also contribute to better reconstructing (photometrically) complex spatially varying BRDF and to improve the efficiency of single-image SVBRDF-based rendering methods (see [51]). In this context, it could be interesting to augment the datasets with synthetic data [52,53,54], for which we can control the input BRDF.

## Figures and Tables

**Table 1:** Viewing and illumination conditions of selected views from KTH-TIPS2 [42] dataset.

View	Viewing Direction	Illumination Direction
View1	Frontal	Frontal
View2	22.5° left	Ambient
View3	Frontal	45° from top
View4	22.5° right	Ambient
View5	Frontal	45° from side
View6	Frontal	Ambient
View7	22.5° right	Frontal
View8	22.5° left	45° from side
View9	22.5° right	45° from top
View10	22.5° left	45° from top
view11	22.5° right	45° from side
view12	22.5° left	Frontal

**Table 2:** Viewing and illumination condition for selected views from the UJM-TIV dataset shown in Figure 5.

View	Viewing Direction	Illumination Direction
View1	90°	90°
View2	90°	45°
View3	90°	20°
View4	60°	65°
View5	60°	20°
View6	30°	90°
View7	90°	65°
View8	60°	45°
View9	60°	90°
View10	30°	20°
View11	30°	45°
View12	30°	65°
View13	10°	90°
View14	10°	20°
View15	10°	45°
View16	10°	65°

**Table 3:** Model accuracy of single branch network with KTH-TIPS2 and UJM-TIV when considering all the views.

Train Data	Test Data	Val. Accuracy
KTH-TIPS2 Train	KTH-TIPS2 Test	80.00
UJM-TIV Train	UJM-TIV Test	55.26

**Table 4:** Model accuracy of single-view and multi-view learning on KTH-TIPS2.

Train Data	Test Data	Single-View Accuracy	Multi-View Accuracy	Improvement (%)
view1, view2	view1, view2	56.90	68.53	+29.76
view3, view4	view3, view4	60.34	67.24	+10.26
view5, view6	view5, view6	56.91	71.98	+20.94
view7, view8	view7, view8	39.66	47.41	+16.35
view9, view10	view9, view10	34.48	64.22	+46.31
view11, view12	view11,view12	37.93	67.24	+43.59

**Table 5:** Model accuracy of single-view and multi-view learning on our UJM-TIV dataset.

Train Data	Test Data	Single-View Accuracy	Multi-View Accuracy	Improvement (%)
view1, view2	view1, view2	50.28	79.52	+36.77
view3, view4	view3, view4	60.00	75.29	+20.31
view5, view6	view5, view6	44.48	95.71	+53.52
view7, view8	view7, view8	51.32	96.52	+46.83
view9, view10	view9, view10	65.59	95.29	+31.17
view11, view12	view11, view12	66.63	94.56	+29.54
view13, view14	view13, view14	80.33	89.34	+10.08
view15, view16	view15, view16	53.91	83.78	+35.65

**Table 6:** State-of-the-art model [50] accuracy of single-view and multi-view learning on KTH-TIPS2.

Train Data	Test Data	Single-View Accuracy	Multi-View Accuracy	Improvement (%)
view1, view2	view1, view2	94.7	97.5	+3.0
view3, view4	view3, view4	90.0	96.67	+6.90
view5, view6	view5, view6	90.83	95.83	+5.22
view7, view8	view7, view8	92.50	98.33	+5.93
view9, view10	view9, view10	92.50	95.83	+3.47
view11, view12	view11, view12	90.00	94.17	+4.40

**Table 7:** State-of-the-art model [50] accuracy of single-view and multi-view learning on our UJM-TIV dataset.

Train Data	Test Data	Single-View Accuracy	Multi-View Accuracy	Improvement (%)
view1, view2	view1, view2	100	98.99	−1.02
view3, view4	view3, view4	99.44	100	+0.56
view5, view6	view5, view6	99.85	100	+0.15
view7, view8	view7, view8	99.88	100	+0.12
view9, view10	view9, view10	99.56	100	+0.44
view11, view12	view11, view12	99.88	100	+0.12
view13, view14	view13, view14	99.80	100	+0.20
view15, view16	view15, view16	98.31	99.58	+1.28

## Data Availability

The UJM-TIV dataset will be publicly available on an open access repository. The UJM-TIV dataset is already available upon request.
